# Searchers adjust their eye-movement dynamics to target characteristics in natural scenes

**DOI:** 10.1038/s41598-018-37548-w

**Published:** 2019-02-07

**Authors:** Lars O. M. Rothkegel, Heiko H. Schütt, Hans A. Trukenbrod, Felix A. Wichmann, Ralf Engbert

**Affiliations:** 1 Department of Psychology, University of Potsdam, Karl-Liebknechtstraße 24/25, 14476 Potsdam, Germany; 2 Neural Information Processing Group, University of Tübingen, Sand 6, 72076 Tübingen, Germany; 3 Max Planck Institute for Intelligent Systems, Max-Planck-Ring 4, 72076 Tübingen, Germany

## Abstract

When searching a target in a natural scene, it has been shown that both the target’s visual properties and similarity to the background influence whether and how fast humans are able to find it. So far, it was unclear whether searchers adjust the dynamics of their eye movements (e.g., fixation durations, saccade amplitudes) to the target they search for. In our experiment, participants searched natural scenes for six artificial targets with different spatial frequency content throughout eight consecutive sessions. High-spatial frequency targets led to smaller saccade amplitudes and shorter fixation durations than low-spatial frequency targets if target identity was known. If a saccade was programmed in the same direction as the previous saccade, fixation durations and successive saccade amplitudes were not influenced by target type. Visual saliency and empirical fixation density at the endpoints of saccades which maintain direction were comparatively low, indicating that these saccades were less selective. Our results suggest that searchers adjust their eye movement dynamics to the search target efficiently, since previous research has shown that low-spatial frequencies are visible farther into the periphery than high-spatial frequencies. We interpret the saccade direction specificity of our effects as an underlying separation into a default scanning mechanism and a selective, target-dependent mechanism.

## Introduction

One of the most important everyday tasks of our visual system is to search for a specific target. Whether the task is to find a fruit amongst leaves, detect a dangerous animal or find relatives in a crowd of people, visual search has always been essential for survival. How the brain performs visual search tasks has been subject to a vast amount of research and, consequently, a number of comprehensive theories have been proposed^1–3^. However, most studies concerning visual search have been conducted on so-called search arrays, where targets and distractors are presented on a homogeneous background. While results from these highly controlled studies are very useful for understanding the basic nature of visual search, many do not take eye movements into account, although eye movements play an important role in real world search behavior^4–6^.

When searching on a complex background, saccades—fast ballistic eye movements—are executed about three to four times per second to increase the probability of finding a target. It has been shown in many studies that the search target strongly influences saccade target selection of eye movements when searching through natural scenes. Object-scene consistency^7–9^, scene context^10–13^ as well as low-level features^14^ of the target influence where observers fixate. Thus, a top-down search template of the target appears to guide gaze during scene exploration^2,14^. Correlations between the visual properties of target-related search templates and fixated image patches exist, but do not completely explain eye-movement behavior in visual search on complex backgrounds. Najemnik and Geisler^15,16^ showed that human observers do not simply move their eyes to positions which maximally resemble the target but rather apply a strategy which takes the visual degradation towards retinal periphery into account. They argue that observers sample as much relevant information as possible with a minimal number of eye movements, which they call the optimal eye-movement strategy in visual search. Thus, it seems useful for the visual system to adapt eye-movement strategies according to the target’s visibility in the periphery. Target visibility depends on retinal eccentricity^17^ and its interaction with many factors such as spatial frequency^18^ and contrast^19,20^.

To investigate whether target features not only influence where participants look at (fixation locations) but also how they search (saccade amplitudes and fixation durations), we let participants search natural scenes for artificial targets with different low-level features. Although one might suspect that different targets lead to different saccade amplitudes and fixation durations, to our knowledge no one has yet provided empirical evidence to answer this question. It is rather important for models of eye movement control to know whether, how fast, and how accurately human observers change search strategies contingent on the target they search for. To explicitly compare targets of different spatial frequency on various backgrounds, we used artificial targets instead of real-world objects in this study. Furthermore, we used scenes instead of plain backgrounds or search arrays because (i) we are interested in real-world search behavior and not search under highly controlled conditions, (ii) to gain knowledge to improve dynamical models of saccade generation in natural scenes^21,22^, (iii) because the grid-like nature of search displays severely constrains the sensible set of saccade directions and amplitudes and (iv) because plain backgrounds often lead to pop-out effects which drastically reduce the amount of eye movements within each trial. Thus, in this study we are specifically interested in adjustments of dynamical measures of eye movements rather than typical measure variables of visual search such as a target’s detectability or search times.

**Figure 1:**
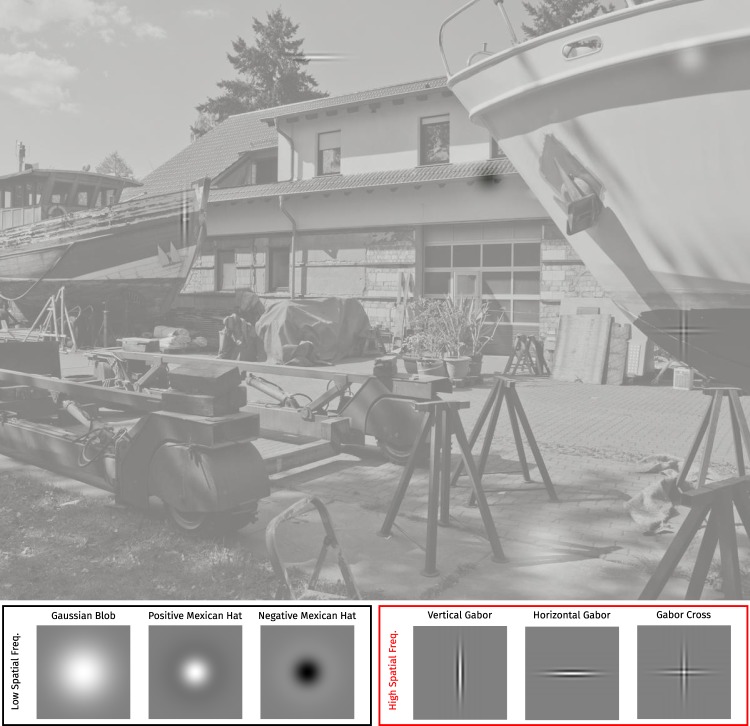
Illustration of the task. Subjects were asked to search for one specific target for a block of 25 trials, each overlayed over natural scenes like this one. In this image all 6 targets are hidden twice as large and at higher contrast than in the experiment, to make them visible despite the small image. In the actual experiment only one target was hidden per image and the image was shown much larger. The bottom panels show the 6 targets we used. The frames around the targets mark which frequency category they belong to.

In our study, observers searched in each of 8 consecutive sessions for 6 targets of varying spatial frequency content and, in the case of high-spatial frequency, orientation (vertical and/or horizontal; see Fig. 1). Each session contained one block per target. Each block consisted of one repetition of the same 25 images. Target type was specified in advance to each block, to provide a search template. In one session (Session 7), targets were chosen randomly for each trial and target type was unknown prior to a trial.

To assure that the difficulty of finding the different targets was not too variable and that detection rates were neither too high nor too low, we conducted a pilot study beforehand and adjusted the targets’ contrast, such that detection rates were predicted at 75–80% for each target. In this pilot study we only used 3 of the six targets for economic reasons.

If dynamical aspects of eye movements are indeed adapted to the search target in a useful way, saccade amplitudes should be larger during search for low-spatial frequency targets, since previous research has shown that low-spatial frequencies can be detected further into the periphery than high-spatial frequencies^18,23–26^. Additionally, fixation durations should be shorter for high-spatial frequency targets, since our high-spatial frequency targets are detected easier if they fall into the fovea than our low-spatial frequency targets^27^. Another reason to prolong fixation durations for low-spatial frequency targets is that low-spatial frequency targets can be perceived from further away, and the size of the window in which targets can be detected increases with longer stimulus presentation^28^.

Thus, we expected a search behavior with small saccade amplitudes and short fixation durations when participants search for high-spatial frequency targets and a search behavior with larger saccade amplitudes and longer fixation durations when participants search for low-spatial frequency targets.

## Results

**Figure 2:**
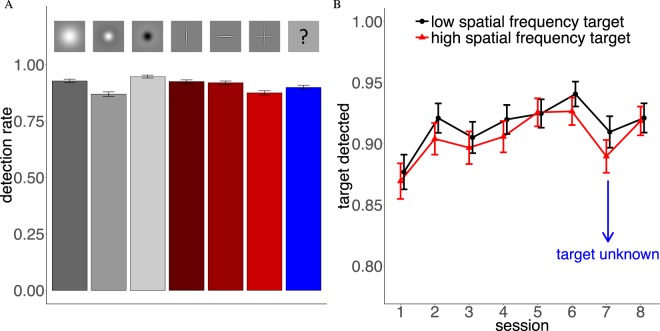
(**A**) Detection rate for the 6 targets. Red bars are high-spatial frequency targets and gray bars low-spatial frequency targets. The blue bar captures all trials where target type was unknown prior to the trial. (**B**) Average detection rate of the three low and high-spatial frequency targets throughout the 8 experimental sessions. In Session 7 target type was unknown prior to a trial.

**Figure 3:**
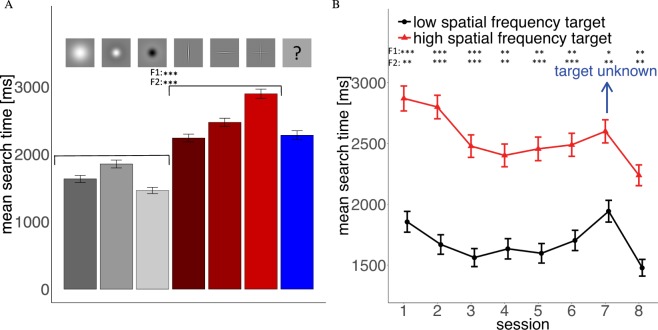
(**A**) Search times for the 6 targets. Red bars are high-spatial frequency targets and gray bars low-spatial frequency targets. The blue bar captures all trials where target type was unknown prior to the trial. (**B**) Average search times for the three low and high-spatial frequency targets throughout the 8 experimental sessions. In Session 7 target type was unknown prior to a trial.

**Figure 4:**
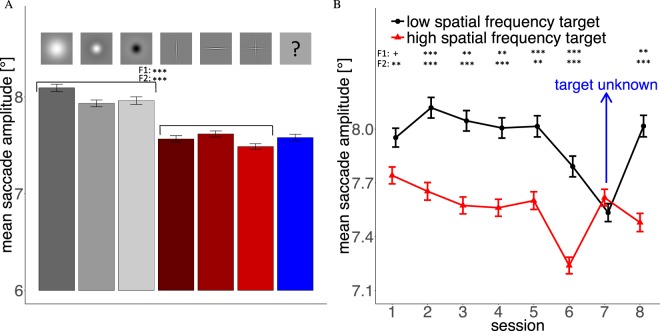
(**A**) Mean saccade amplitude for the 6 different targets. Red bars are high-spatial frequency targets and gray bars low-spatial frequency targets. The blue bar captures all trials where target type was unknown prior to the trial. (**B**) Average saccade amplitude of the three low and high-spatial frequency targets throughout the 8 experimental sessions. In Session 7 target type was unknown prior to a trial.

**Figure 5:**
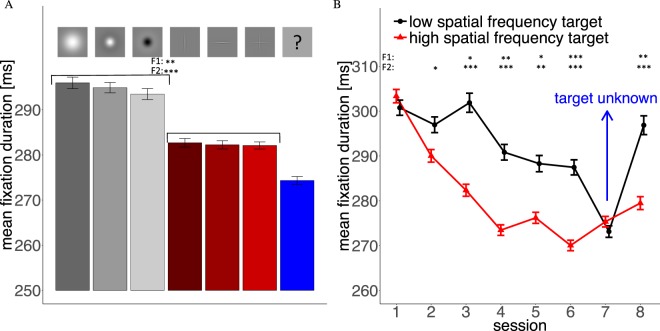
(**A**) Mean fixation durations for the 6 different targets. Red bars are high-spatial frequency targets and gray bars low-spatial frequency targets. The blue bar captures all trials where target type was unknown prior to the trial. (**B**) Average fixation duration of the three low and high-spatial frequency targets throughout the 8 experimental sessions. In Session 7 target type was unknown prior to a trial.

**Figure 6:**
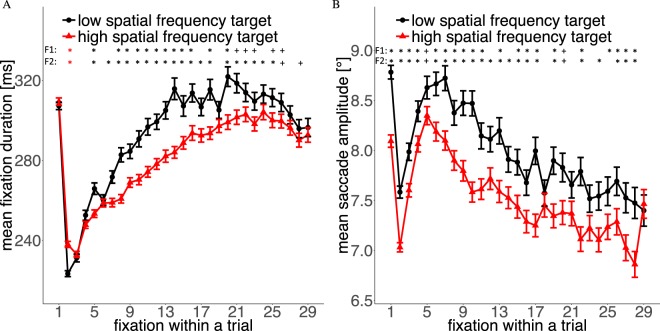
Temporal evolution of (**A**) mean fixation duration and (**B**) saccade amplitude for the two targets types throughout a trial.

**Figure 7:**
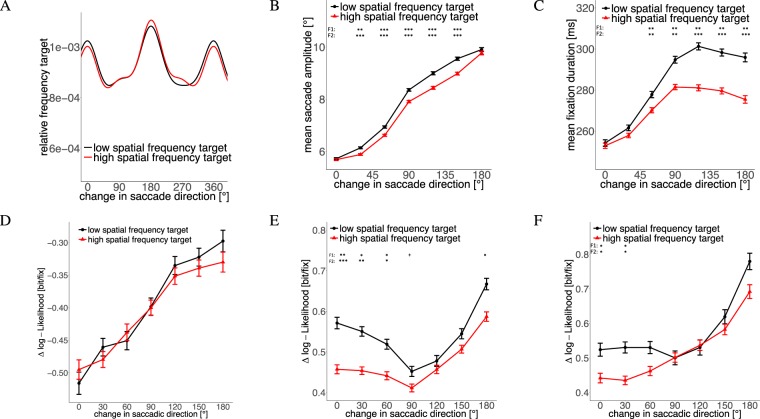
(**A**) Density distribution of change in saccadic direction. Influence of change in saccadic direction on (**B**) successive saccade amplitude, (**C**) fixation duration between the two saccades, (**D**) DeepGaze saliency of successive saccade target, (**E**) empirical density of successive saccade target and (**F**) empirical density of successive saccade target only for saccades between 3 and 8 dva.

We analyzed eye movement data from our experiment for search accuracy and search speed as control measures first. We then computed average fixation durations, average saccade amplitudes and effects of changes in saccadic direction to see if searchers adjust dynamics of their eye movements due to the target they search for. All variables were investigated separately for the different search targets. Bar plots (left side of Figures 2–5) represent results for each of the 6 targets and the results for all targets combined in Session 7, when target type was unknown prior to each trial. Line graphs in Figures 2–7 show comparisons between the three low-spatial frequency targets (Gaussian Blob and positive/negative Mexican hat, black line) and the three high-spatial frequency targets (vertical, horizontal bar and cross, red line; cf. Fig. 1, bottom panels) throughout the course of the 8 experimental sessions. Error bars in the graphs are the standard error of the mean. For statistical tests of significance, we computed F1- and F2-ANOVAs aggregated over id and image, respectively. After aggregation, we computed an ANOVA for each comparison with the ezANOVA command from the ez-package for the R language of statistical computing^29,30^ with spatial frequency as the independent variable. Significance signs in Figures 3–7 refer to differences between low- and high-spatial frequency targets (+*p* < 0.1, *p < 0.05, **p < 0.01, ***p < 0.001). In all figures we report significances for both types of data aggregation. The first line of significances refers to an aggregation on the level of id (F1) and the second on the level of images (F2). When comparing for each independent fixation number within a trial (Fig. 6) we only distinguish between *p* < 0.1 and *p* < 0.05 to avoid a cluttered figure. Note that some tests were not significant when the data was aggregated on the level of id. This is not surprising since each spatial frequency condition contained only data from 10 participants. Reported p-values were not adjusted for multiple comparisons, since hypotheses were directed and effects were stable across multiple comparisons. Note, applying a Bonferroni correction is less sensitive but does not change the overall pattern of results.

### Task performance

#### Detection Rate

Performance of our group of 10 participants (see Methods) is characterized by similar detection rates (Hits/Misses) for the different targets throughout the whole experiment (Fig. 2A). The lowest detection rates were observed for the positive Mexican hat and the high-spatial frequency cross (both 83%) and the highest rate for the negative Mexican hat (92%). The overall rate of false alarms was very low (3.44% of target absent trials). Over the course of the experiment (Fig. 2B), the detection rate for both low and high-spatial frequency targets increased. No clear difference between the groups of high-spatial frequency and low-spatial frequency targets was observed. In Session 7, when target type was unknown prior to the trial, detection rates dropped for both target types but performance was still better than in the first experimental session. Thus, target detectability shows limited variability between different target types and detection rates turned out to be significantly above chance. Additionally, finding the target was difficult enough to prevent ceiling effects. Although some small differences between detection rates were visible, there was no significant overall difference between the groups of high- and low-spatial frequency targets. These results show that differences in other eye movement parameters cannot easily be attributed to the difficulty of finding the target.

#### Search Time

Mean search times (Fig. 3A) were more variable between the targets than the detection rates. Participants were on average faster at finding low-spatial frequency targets than high-spatial frequency targets. Participants were fastest at finding the negative Mexican hat and slowest at finding the high-spatial frequency cross. Search time decreased over the 8 sessions. The first 3 sessions showed a clear training effect and afterwards a plateau was reached (Fig. 3B). In Session 7 (target unknown) search times increased but high-spatial frequency targets were still detected faster than in the first session, indicating that search training compensated for loss of guidance in this case, which was also visible in detection performance. Although search times are not our main interest in this study, these results indicate that our low-spatial frequency targets can be seen from further away than the high-spatial frequency targets, because lower search times indicate that fewer saccades and fixations were necessary to find these targets.

### Scanpath properties

#### Saccade Amplitudes

Analyses of the saccade amplitudes throughout our experimental sessions (Fig. 4) showed three clear results: (i) Mean amplitudes were greater for low than for high-spatial frequency targets, (ii) this difference was already indicated in the first session, persisted significantly throughout all later sessions, and (iii) vanished when target type was unknown prior to a trial. Searching for the Gaussian blob led to the largest mean saccade amplitudes (Fig. 4A). Overall, low-spatial frequencies produced larger saccade amplitudes, indicating that search strategy was adjusted to the visibility of the targets into the periphery. In addition to these interpretable results, saccade amplitudes dropped for all targets in Session 6. As this session did not differ from any of the previous sessions in any experimental parameters and no other parameters changed, the effect of this drop might be due to coincidental fluctuations across several participants. Indeed subjects show some variance in saccade length from session to session, which affects all targets, and 4 subjects produced relatively large drops in saccade amplitude in Session 6 but still produced significant differences between high- and low-spatial frequency targets.

#### Fixation Durations

The pattern for mean fixation durations (Fig. 5) was similar to the pattern of saccade amplitudes: (i) The three low-spatial frequency targets led to a search strategy with longer fixation durations, (ii) this difference in fixation durations needed between one and two training session to be established, but afterwards persisted throughout the other sessions, and (iii) vanished when target type was unknown prior to a trial. Fixation durations decreased throughout the experiment, thus mean fixation durations were rather short in Session 7, when the target was unknown prior to each trial (Fig. 5B). Again, the search strategy was adjusted according to the spatial frequency of the targets in a useful way, since it takes longer for low-spatial frequency targets to be detected when they fall into the fovea.

#### Time-course During a Trial

In the sequence within a trial, mean fixation durations increased and mean saccade lengths decreased (except for the first fixations/saccades, which were influenced by the experimental design and the central fixation bias, see Methods; Fig. 6). This behavior is known as the coarse-to-fine strategy of eye movements^31,32^. However, the effect of target spatial frequency already occured after the second saccade for saccade amplitudes and lasted for the rest of the trial. In the case of fixation durations, it took some time (about 6–7 fixations) until the difference was stable but then persisted at least for the next 9–10 fixations. Thus, participants displayed different coarse-to-fine strategies for low and high-spatial frequency targets.

#### Change in Saccadic Direction

Although we did not have a hypothesis about the interaction of saccade direction and visual search target, we included corresponding post-hoc analyses, since angles between successive saccades follow a very characteristic distribution in scene-viewing experiments^33–35^ and interact strongly with saccade amplitudes and fixation durations^36,37^. Saccades, which maintain direction (which we will denote as *saccadic momentum* saccades in the following^34^) typically have small amplitudes and preceding fixation durations are short. Saccades after a 180 degree change in direction (or *return saccades*) usually have large amplitudes and fixations, which precede this return saccade, last rather long.

No apparent difference was found between the distributions of intersaccadic angles for low and high-spatial frequency targets (Fig. 7A). For both target types, most saccades either maintain direction or completely reverse direction (see also^33–35^). As has been described previously^36^, we observed an increase in saccade amplitude as a function of preceding change in saccade direction. Figure 7B shows that saccades, which maintained direction from the previous saccade (0 degree change), were equally large for both target types. Saccades which were preceded by a change in direction differed in saccadic amplitude, except for complete turns in direction (180 degree change). An influence of the angle is even more evident if we look at fixation duration differences between the two target types (Fig. 7C). Again, fixation durations increased for large changes in saccade direction. However, compared to previous results^36^, the increase in our experiment was not linearly but reached a plateau for large changes in saccade direction and even decreased slightly for complete return saccades. Again, we see that saccades which maintain direction (0 degree change) show no difference for fixation durations between the two target types. For saccades which change direction, the fixation durations differ between the two target types. The fact that both, saccade amplitude and fixation duration do not differ for saccades without a change in direction led us to the hypothesis that these saccades are less selective than other saccades and belong to a default scanning mechanism.

To further investigate this hypothesis, we compared the saccadic landing points for different changes in saccade direction in terms of empirical density and visual saliency. The empirical density maps were computed with the SpatStat package of the R language for statistical computing^30,38^. For visual saliency, we used the DeepGaze II model^39^, which is the currently highest ranking saliency model on the MIT saliency benchmark^40^. The fixations were evaluated in terms of their likelihood under the Deep Gaze II model or the empirical density compared to a uniform distribution (see^22,41^ for further elaboration). Values above zero indicate improvements compared to a uniform distribution, negative values represent predictions below chance.

Figure 7D,E show that saliency and empirical density, respectively, depended on the previous change in saccade direction. Saccade targets were most salient (Fig. 7D) and visited more by all other participants (Fig. 7E) if the previous saccade had a large change in direction (180 degree). Saliency values increased continuously with larger changes in saccade direction. Empirical density of the saccade targets was highest for return saccades (180 degree) but lowest for saccades with a left or right turn (90 degree) from to the previous saccade. This did not match our hypothesis that the least selective saccades are saccades which maintain direction (0 degree change). However, most saccades which maintain direction are rather short, and short saccades often land at highly interesting positions, because they contain corrective saccades. Thus, we analyzed the empirical density with respect to the preceding change in direction for different saccade amplitudes separately. Figure 7F shows the empirical density with respect to previous change in saccade direction only for saccades between 3 and 8 degrees of visual angle. Removing the rare large saccades and small corrective saccades led to the smallest empirical density for saccades without a change in direction, as hypothesized for a default scanning mechanism. If we conduct this analysis for one degree bins of saccade amplitude sizes separately, the increase of empirical density for an increasing change in saccade direction is evident for all amplitudes between 2 and 11 degree. Larger saccades show a rather noisy distribution and smaller saccades a rather constant (and very high) value, independent of the previous change in saccade direction.

Although visual saliency depended on the change in direction (Fig. 7D), all fixations are predicted below chance by the Deep Gaze II model (the difference in log-likelihood compared to a uniform prediction was negative). This finding is in good agreement with the notion that visual saliency does not predict fixation locations in visual search above chance^42,43^. It is important to note, however, that Deep Gaze II specifically predicts higher-level objects and that our targets were low-level Gabors. Therefore, one might expect that a more low-level saliency model would predict fixations better. In another paper, however, we report results that in this experiment low-level saliency models fail to predict fixation locations with high accuracy^43^.

### Spatial frequency spectra of fixated locations

Earlier analyses of eye movements during visual search reported similarities between the fixated locations and the target and, consequently it was assumed that such relationships could be exploited for the prediction of fixation locations^14^. Thus, we investigated whether a corresponding difference between fixated and non-fixated image locations exists in our data. As fixated locations, we extracted patches around the fixation locations and compared them to control patches extracted from the same locations in a different image from the stimulus set^44,45^ (see Methods).

**Figure 8:**
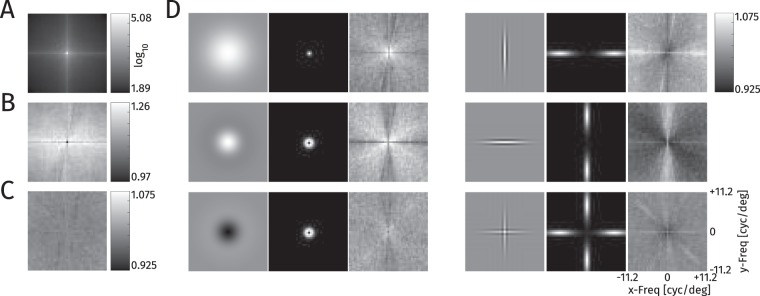
Analysis of spatial frequency amplitude spectra at fixated locations. (**A**) Grand average spectrum over all fixated patches. (**B**) Spectrum from A divided by the average spectrum at control locations. The value at 0 frequency is 0.97, all other values are in the range of [1.09, 1.26] (**C**) Average spectrum for fixations when the target is unknown, plotted as for known targets in D. (**D**) Triples for each target: The target at 100% contrast against a gray background, the amplitude spectrum of the target and the average amplitude spectrum at fixation locations divided by the average over all targets. The color range from black to white for the third plot is always [0.925, 1.075].

To compare the fixated patches for the different targets, we analyzed the spectra of the patches (Fig. 8; see Methods). As displayed in Fig. 8A, the average spectrum of a fixated patch looks much like the spectrum of any image patch with a clear $\frac{1}{f}$ decline in spatial frequency content and a preference for horizontal and vertical structure. As these strong effects hide all other effects, all other spectra are divided by the spectra of the comparison patches for display.

The overall spectrum of fixated patches shows increased power for all frequencies and orientations (Fig. 8B) compared to a random image patch, indicating that fixated patches have more contrast than non-fixated patches. The unknown target condition (Fig. 8C) produces no clear deviation from the average over the conditions with known target. Searching for a specific target produces a slight bias of the fixated image patches towards being more similar to the spectrum of the target (Fig.8D). The deviations of the single targets from the grand average are all smaller than 5%, however, while the variance over patches is substantial ($\frac{SD}{M}\in \mathrm{[78.65 \% ,\; 161.03 \% ]}$, average = 91.10%).

While these results indicate a bias towards image patches, which have similar spectrum to the target, differences in the range of 0.1 standard deviations are certainly too small to infer the fixation category from the spectrum. Thus, the only distinction, which might provide some predictive value, is the generally increased contrast at fixated locations in general.

### Target difficulty

**Figure 9:**
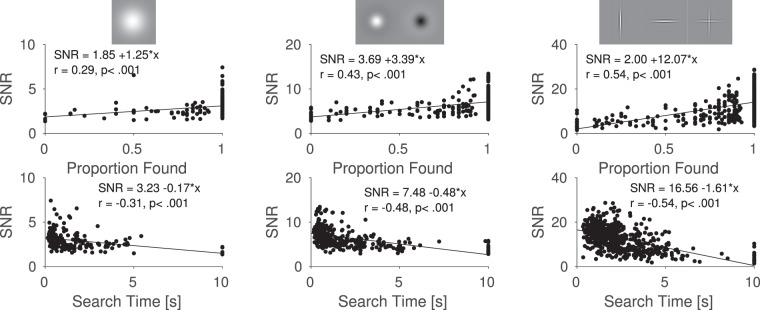
Signal to Noise Ratio from an early vision model for all target-background combinations^27^. Search times and detectability are correlated, when separating analysis between target types.

Since we placed the targets on different, pseudorandom positions in the images, it was - by chance - sometimes easy and sometimes hard to find them, although the different targets were placed in the same positions within each image (see Methods). A direct measure of how difficult it was to find a target, is the time it took participants to find the target. As a computational measure, we used a recently published early vision model for images, which computes a signal-to-noise-ratio (SNR) of the target on the background for all target patches^27^, (i) to evaluate whether the model can predict search behavior on natural scenes and (ii) to obtain a measure of visibility for each of the targets at each position. A glance at Figure 9 indicates that both, detectability and search time were correlated with the predicted SNR from the early vision model. The computed SNR by the early vision model thus predicts search difficulty. The model computes SNRs for foveal vision. Note however, that the high-spatial frequency targets have higher SNRs than the low-spatial frequency targets and are thus on average easier to see over backgrounds, when looked at in foveal vision (different scales of y-axis in Fig. 9). Nonetheless, low-spatial frequency targets were found significantly faster than high-spatial frequency targets, arguing that the periphery and eye movements play a highly important role in visual search^46^.

## Discussion

We studied visual search for artificial low and high-spatial frequency targets in natural scenes and found that fixation durations and saccade amplitudes depend on the low-level properties of the search target. The different influences of a target on these basic eye-movement characteristics are part of a top-down search strategy, since differences between targets disappeared immediately, as soon as participants did not know which target to search for. Additionally, differences between target types also occurred when the target was absent but participants were told which target to look for. Our findings imply that humans adjust their basic search behavior to the target they look for. In our study, fixation durations and saccade amplitudes were longer for low-spatial frequency targets. Previous research has shown that detectability of targets in the periphery depends on spatial frequency^18^ and fixation duration^28^. Increasing fixation durations thus leads to a larger window of detectability and low-spatial frequency targets can generally be detected from further away. For high-spatial frequency targets, participants decreased their fixation durations strongly over time. Decreasing fixation durations and thus increasing frequency of fixations for high-spatial frequency targets are a useful search strategy because high-spatial frequency targets usually (i) cannot be detected from far away and (ii) have a higher SNR computed by our model, when looked at directly (see Fig. 9). These results show that dynamical eye movement characteristics are adjusted when participants look for different targets. How close to optimal the subjects’ search strategies are, and how exactly subjects switch or adjust their search strategies we cannot, unfortunately, answer from the data reported herein.

A recently published model of early vision^27^ was used to predict target difficulty. Separately looking at the results for the different targets produced promising results. We found high correlations between a targets signal-to-noise-ratio and search times and detectability values. However, the targets themselves had very different signal-to-noise-ratios, which were not reflected in search times. One possible reason that the model fails at predicting search times between different target types is, that only foveal input is modeled. The fact that low-spatial frequency targets are found faster than high-spatial frequency targets, although they have a lower SNR, shows that the periphery plays a strong role when searching for artificial targets on complex backgrounds.

Analyses of the fixation locations demonstrate that searchers slightly adjust where they look to depending on the target and confirming earlier reports^2,14^. However, the influence of the target on fixation locations (investigated by comparing spatial frequency spectra) is rather small, agreeing with the notion that participants do not merely look at positions which mostly resemble the target^15^, but take their peripheral vision into account.

Additional post-hoc analyses of the changes in saccade direction and its dependence on further viewing behavior revealed interesting results. Saccades, which generated strong changes with respect to previous scanpath direction, landed at locations with higher empirical fixation probability (when removing small corrective saccades from the analysis) and visual saliency, and were influenced by target properties, except for saccade amplitudes after a 180 degree change in direction. Saccades which maintained direction were not influenced by the search target and corresponding endpoints had low fixation density and low saliency values. For the control of fixation durations, our current results lend support to the concept of mixed control^47,48^, meaning that the visual input as well as some independent time-keeper influences when a saccade is generated. We thus assume that some saccades which maintain direction from the last saccade are not exhibited through volitional control or triggered by attentional capture but are movements generated by a default program of the motor system. We interpret our results as an indication for two different ways of searching, a selective search and a default scan, which primarily moves the eyes forward in one direction. These findings agree with a study by Bays and Husain^49^, who reported that return saccades are generally inhibited and only executed if a saccade target is highly interesting while forward saccades are facilitated and more frequent than a random, memoryless control mechanism would predict.

Eye movements play a substantial role for visual search in natural scenes and are at least partially under top-town control. However, there also seems to be a default scanning mechanism, which continues to move the eyes in the previous saccade direction and is not adjusted to needs of the target-template. This default scan might simply be the result of an evolutionary program to facilitate foraging^50^. Thus, our results are consistent with at least two mechanisms controlling eye movements under natural search conditions, which is important for dynamical models of scanpath generation^21,37,51^.

## Methods

We generated 6 different low-level targets with different orientation and spatial frequency content (Fig. 1):

A *Gaussian blob* with a standard deviation of 0.4° of visual angle. This is an isotropic stimulus, which is a Gaussian in spatial frequency as well (with a standard deviation of *σ*_*f*_ = 0.3979). A *positive Mexican hat*, the difference between a Gaussian with a standard deviation of 0.2° and a Gaussian with standard deviation 0.4°. This stimulus is isotropic and has a peak frequency of roughly $0.7\frac{cyc}{deg}$. A *negative Mexican hat*, the negative of the positive Mexican hat, which has exactly the same spatial frequency spectrum. A *vertical Gabor*, the product of $8\frac{cyc}{deg}$ vertical cosine centered at the origin and a Gaussian with standard deviations of 0.06° and 0.32° in x and y direction. In frequency space this stimulus is strongly oriented and has a relatively broad frequency peak at $8\frac{cyc}{deg}$. A *horizontal Gabor*, the same as the vertical Gabor but oriented horizontally. A *Gabor cross*, the sum of the two Gabors, each at half the contrast.

All stimuli but the Gaussian blob were near zero mean and all stimuli were normalized to have an amplitude of 1, i.e. *max*(*abs*(*T* )) = 1.

To embed the targets into the natural images, we first converted the image to luminance values based on a power function, fitted to the measured luminance response of the monitor. We then combined this luminance image *I*_*L*_ with the target *T* with a luminance amplitude *αL*_*max*_, fixed relative to the maximum luminance displayable on the monitor *L*_*max*_ as follows:

1 $${I}_{fin}=\alpha {L}_{max}+\mathrm{(1}-2\alpha ){I}_{L}+\alpha {L}_{max}T.$$

We rescaled the image to the range [*α*,(1−*α*)]*L*_*max*_ and then added the target with a luminance amplitude of *αL*_*max*_, such that the final image *I*_*fin*_ never left the displayable range. We then converted the image *I*_*fin*_ back to [0,255] grayscale values by inverting the fitted power function.

### Target locations

For placement of the targets we lay a grid of 4 × 2 rectangles over each image. Within each rectangle, we chose a random position for each target and image, which was at least 100 pixels away from the border, such that the target was not cut off at any side. The original plan was to present each target at each position in each image once over the eight sessions of one observer. Unfortunately, a bug in the experimental code led to a random choice of the target location instead, but we sampled only among the 8 possible locations for each target-image combination. Most target-position-image combinations appeared between 6 and 10 times (10 participants and about 20% target absent trials, mean = 7.8) and none was present more than 16 times. We are rather certain that participants could not remember the position-target-image combinations over 1200 trials. Overall, about a third of the experimental trials included combinations that had already been presented before. We conducted all our analyses without the trials where a target-image-position combination had already been seen by a participant and no differences were observed in any of the outcome measures. Furthermore, no participant mentioned noticing anything like repeating target positions.

### Experiment

#### Stimuli

As stimulus material we used 25 images taken by L.R. and a member of the Potsdam lab with a Canon EOS 50D digital camera (max. 4752 × 3168 pixels). The images were outdoor scenes without people, animals or written words present. Most images had parts with a lot of high-spatial frequency content (grass or woods) and parts with no high-spatial frequency content (sky or empty street). They were all taken on a bright sunny day in the summer.

#### Stimulus Presentation

Stimuli were presented on a 20-inch CRT monitor (Mitsubishi Diamond Pro 2070; frame rate 120 HZ, resolution 1280 × 1024 pixels; Mitsubishi Electric Corporation, Tokyo, Japan). All pictures were reduced to a size of 1200 × 960 pixels. For the presentation during the experiment, images were displayed in the center of the screen with gray borders extending 32 pixels to the top/bottom and 40 pixels to the left/right of the image. Images covered 31.1 degree of visual angle in the horizontal and 24.9 degree in the vertical dimension.

#### Participants

We recorded eye movements from 10 human participants (4 female) with normal or corrected-to-normal vision in 8 separate sessions on different days. 6 participants were students from a nearby high school (age 17 to 18) and 4 were students at the University of Potsdam (age 22 to 26). The work was carried out in accordance with the Declaration of Helsinki. Informed consent was obtained for experimentation by all participants. According to the standards of Deutsche Forschungsgemeinschaft (German Research Foundation) and German Society for Psychological Research, ethics committee approval was not required for this study.

#### Procedure

Participants were instructed to position their heads on a chin rest in front of a computer screen at a viewing distance of 70 cm. Eye movements were recorded binocularly using an desktop mounted Eyelink 1000 video-based-eyetracker (SR-Research, Osgoode/ON, Canada) with a sampling rate of 1000 Hz. Participants were instructed to search a target for the upcoming 25 images. Before each block of 25 images, the target was presented on an example image, marked by a red square. Each session consisted of 6 blocks with 25 images with the 6 different targets. The 25 images were always the same images.

Overall, 10 participants searched 6 targets on 25 images in 8 sessions, thus we collected data of 12000 search trials. Target absent trials made up between 3 and 7 for each block of 25 images (~20%).

Trials began with a black fixation cross presented on gray background at a random position within the image borders. After successful fixation, the image was presented with the fixation cross still present for 125 ms. This was done to assure a prolonged first fixation to reduce the central fixation tendency of the initial saccadic response^52,53^. After removal of the fixation cross, participants were allowed to search the image for the previously defined target for 10 s. Participants were instructed to press the space bar to end the trial, once a target was found.

At the end of each session participants could earn a bonus of up to 5 € additional to a fixed 10 € reimbursement, depending on the number of points collected. Participants earned 1 point for each correctly identified target. If participants pressed the bar although no target was present, one point was subtracted.

#### Data Preprocessing and Saccade Detection

For saccade detection we applied a velocity-based algorithm^54,55^. This algorithm marks an event as a saccade if it has a minimum amplitude of 0.5 degree and exceeds the average velocity during a trial by 6 median-based standard deviations for at least 6 data samples (6 ms). The epoch between two subsequent saccades is defined as a fixation. All fixations with a duration of less than 50 ms were removed from further analysis since these are largely glissades^56^. The number of fixations for further analyses was 166,903. The table of fixations as well as the images from the experiment are publically available and can be downloaded under 10.17605/OSF.IO/CAQT2.

### Fixation locations analysis

#### Empirical Density and Saliency at Saccadic Endpoints

To estimate empirical fixation densities, we used kernel density estimation as implemented in the R package SpatStat (version 1.51–0). To estimate the bandwidth for the kernel density estimate we used leave one subject out cross-validation, i.e. for each subject we evaluated the likelihood of their data under a kernel density estimate based on the data from all other subjects repeating this procedure with bandwidths ranging from 0.5 to 2 degrees of visual angle (dva) in steps of 0.1 dva. We report the results with the best bandwidth chosen for each image separately. We then took the resulting density value of each saccade target and averaged for the different target types and previous changes in saccade direction. Likelihood values are the average of each fixation position on the map, taken from a grid of 128 × 128 grid cells. The DeepGaze II model^39^ provides a map, where we could simply draw saliency values for each fixation. We again averaged these values for the different target types and previous changes in saccade direction.

For further information on likelihood evaluation of saliency models we refer to^22,41,43^.

#### Spatial Frequency Spectra

To analyze the image properties at fixation locations, we extracted image patches around fixation locations and compared them over targets and to comparison locations. We extracted 79×79 pixel patches (≈2.05 × 2.05 dva), around the fixated pixel, for all fixation locations for which this patch lay entirely inside the image. To obtain comparison patches, we extracted patches at the measured fixations locations shifting the image index by one, i.e., we used the fixations from picture one to extract patches from picture two (and so on), and the fixations from the last picture to extract patches from the first picture, as was done earlier to train saliency models^44,45^.

For our analysis, we converted the patches to luminance using the measured gamma curves of the screen and calculated the spatial frequency spectrum using MATLAB’s fft2 function. Then we calculated the amplitude as the absolute value for each frequency and averaged it over patches within a group to display. To display differences between conditions, we divided the average of one group by the average of the other. To quantify the variability of patches within one condition we divided the standard deviation of amplitudes by the mean value $(\frac{SD}{M})$.
